# Life History of *Passaloecus pictus* Ribaut, 1952 (Hymenoptera, Pemphredonidae)

**DOI:** 10.3390/insects15120928

**Published:** 2024-11-27

**Authors:** Piotr Olszewski, Przemysław Strażyński

**Affiliations:** 1 Faculty of Biology and Environmental Protection, Natural History Museum, University of Lodz, Kilińskiego 101, 90-011 Lodz, Poland; 2 Department of Entomology and Agricultural Pests, Institute of Plant Protection–National Research Institute, Węgorka 20, 60-318 Poznań, Poland; p.strazynski@iorpib.poznan.pl

**Keywords:** bionomics, behaviour, nesting activity, digger wasps, *Passaloecus pictus*, kleptoparasites

## Abstract

The nesting of *Passaloecus pictus* Ribaut, 1952 was observed in Kowalewo Pomorskie (northern Poland). The nests were built in trap nests that were placed on a windowsill facing southwest. The females used resin from *Picea pungens* Engelm., located 5 m away, to build the cell walls. The tunnel consisted of 1–7 cells. The females provided provisions for two to three cells per day. The frequency with which a female brought aphids to a nest cell varied from 1 to 7 min. No males were observed near the nest. A total of 42 cells were examined (consisting of 31 female cells and 11 male cells). On average, a female brought about 33 aphids to the female cells and 21 aphids to the male cells. The prey were *Macrosiphoniella millefolii* (De Geer, 1773), *Dysaphis crataegi* (Kaltenbach, 1843), *Macrosiphoniella persequens* (Walker, 1852) and *Uroleucon achilleae* (Koch, 1855). The nest parasites were *Melittobia acasta* (Walker, 1839). The occurrence of a second generation has been confirmed.

## Simple Summary

*Passaloecus pictus* Ribaut, 1952 are small, black-pigmented predatory wasps whose females prey on aphids using them as food for their larvae and are, therefore, considered very useful from an economic point of view. The prey species observed comprised four species of aphids inhabiting the plants typical of psammophilous grasslands. It was found that *P. pictus* has no particular food specialisation and preys on the aphids existing in the biotope surrounding its nest. The species readily uses wooden bee trap nests to build nests. The species develops two generations per year.

## 1. Introduction

The genus *Passaloecus* Shuckard, 1837, belonging to the family Pemphredonidae [1], is represented in the world fauna by 45 species [2], twelve of which have been recorded in Poland [3]. *Passaloecus* resembles the genus *Diodontus* Curtis, 1834 (which has small spikes on tibia 3 and a pygidial plate) and the genus *Polemistus* de Saussure, 1892 (which has protruding lobes on the sides of the clypeus and the inner edges of the eyes strongly converge downwards). The characteristic features of *Passaloecus* include two submarginal and two discoidal cells on the forewing, a metasomal with a short petiole, and one or more vertical and horizontal punctate grooves on the mesopleuron. These insects mainly choose plant stems, decayed wood and galls as nesting sites [4]. The linearly arranged nest cells are separated by partitions made of conifer resin, sometimes with the addition of tiny pebbles and sand grains brought in from the outside [5]. Many of the cells are usually separated from the entrance to the outside by a hollow space [5]. The nests are divided and plugged with coniferous resin or small grains of earth or pebbles [4]. The egg is laid on the top or side of an aphid prey [4]. All native Polish *Passaloecus* species are black-pigmented and reach a maximum size of 6.5 mm [6]. *Passaloecus pictus* Ribaut, 1952 belong to a group of species with two distinct horizontal and oblique rows of thick punctures on the mesopleuron. The labrum in a female is elongated and light-yellow with concaved walls, whereas in a male it is yellow–brown. Furthermore, the last three antennal segments in a male are devoid of tyloidea [6]. In Poland, a *Passaloecus pictus* was first recorded in the Greater Poland–Kuyavia Lowland [7]. The biology of a *P. pictus* was described by Janvier [8]. Their nest is usually built in the ground in sandy areas, in places with steep slopes or, less often, in dead wood [9]. It consists of a 12–15 cm long burrow and five to eight brood cells. The females catch aphids of the genus *Macrosiphum*. They can store up to 30 prey in a single cell. The egg stage usually lasts three to four days. The larva eats all the prey within a week. It usually overwinters as a prepupa [9]. Pupation occurs several weeks before the first flights begin [9,10]. The objective of the present study is to add to the existing information on their nesting biology, including (1) nesting behaviour, (2) nest structure, (3) prey range, (4) phenology and (5) the accompanying kleptoparasites.

## 2. Materials and Methods

**Figure 1:**
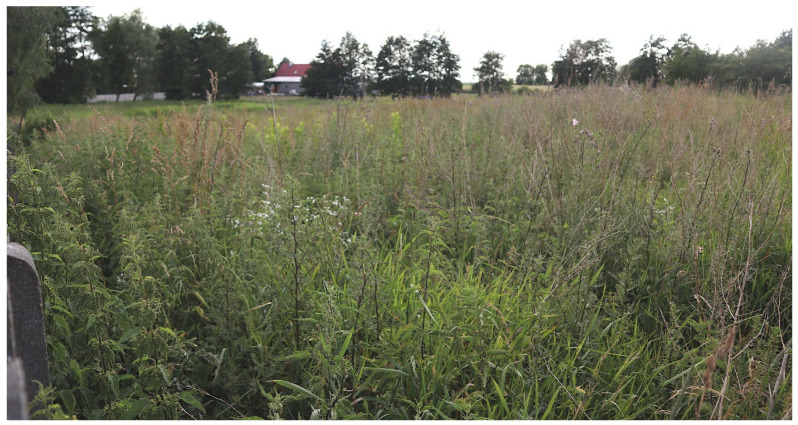
Habitat of *Passaloecus pictus* at the study site.

**Figure 2:**
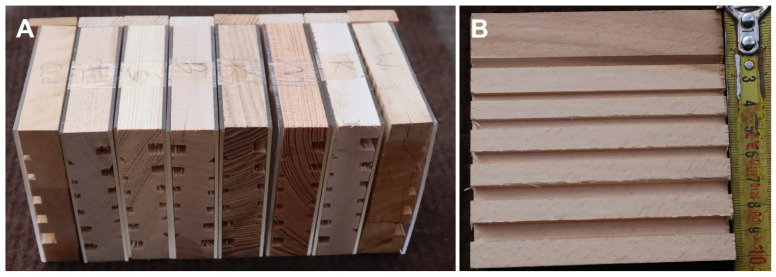
Trap nests: (**A**) front view, and (**B**) side view.

The research was conducted in Kowalewo Pomorskie (53°10′5.8″ N; 18°52′14.6″ E) on a windowsill facing southwest. Observations were made at the nesting site on sunny and warm days (with a temperature of at least 18 °C) from 15 June to 1 August 2024. The nesting site was surrounded by an agricultural wasteland dominated by plants such as *Malva moschata* L., *Hypericum perforatum* L., *Fragaria vesca* L., *Urtica dioica* L., *Lotus corniculatus* L. and *Cirsium arvense* (L.) Scop. (Figure 1). The behaviour of the females, the construction of the nest and the frequency with which the females provisioned the cells were analysed based on the videos recorded using an EOS M50 camera. In addition, a Raynox M-250 macroscopic lens was used for photography and direct observation. To produce the structure of the *P. pictus* nest in the field, we used trap nests constructed from 100 × 25 × 100 mm wooden blocks. There were 65 wooden blocks in total. Each block consisted of six drilled tunnels on each side, with the exception of the five blocks where the tunnels were only on one side. The smallest tunnel at the bottom had a triangular cross-section with a wall width of 2.5 mm, while the other tunnels had a square cross-section with wall widths of 2.5, 3, 4, 6 and 7 mm (Figure 2). The side walls were made of plexiglass, enabling a quick inspection of their interiors. A total of 65 wooden blocks were bound together with adhesive tape. The trap nests were mounted 1.5 m above the ground on a windowsill facing southwest and covered with sheet metal to protect them from rain. The prey of *P. pictus* were placed in 80% alcohol. The nest kleptoparasites were identified based on cultured material. Some specimens of the *P. pictus* imago obtained from the cells and kleptoparasites were deposited in the Natural History Museum of the University of Łódź. The aphids were identified using the relevant identification keys [11,12,13], internet studies [14] and our own collection of aphid preparations. We measured the length and diameter of the brood and vestibular cells using callipers. The total time of development in the field from egg laying to the emergence of an adult (imago) was determined based on a nest inspection.

## 3. Results

**Figure 3:**
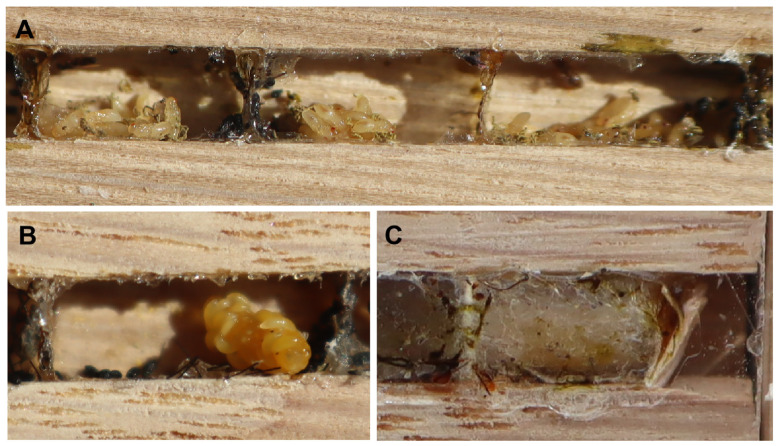
Kleptoparasites of cells: (**A**) pupae of *Mellitobia acasta*; (**B**) larvae of *M. acasta* on the *P. pictus* larva; and (**C**) cuckoo wasp cocoon.

**Figure 4:**
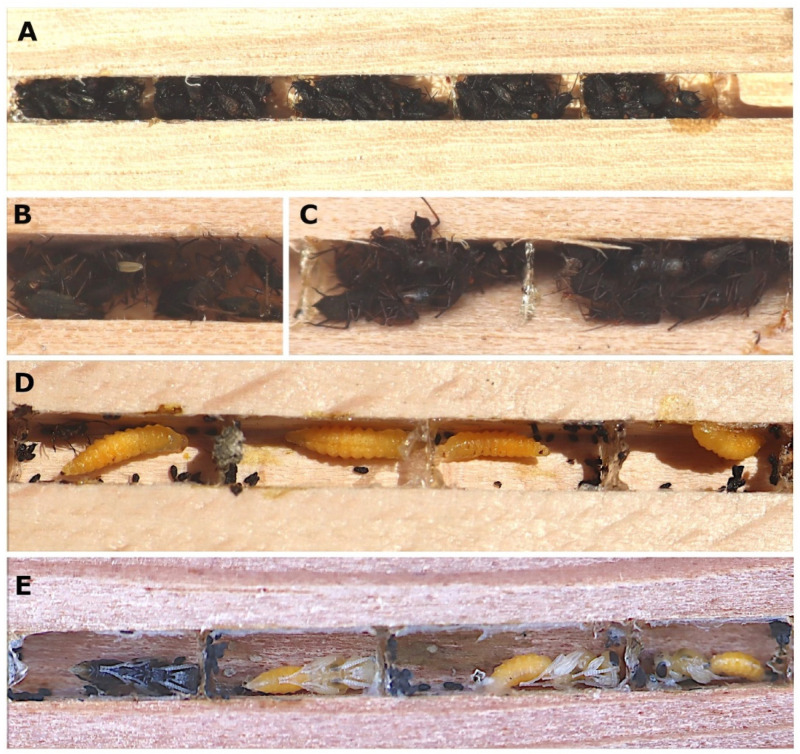
Nests and immature stages of *Passaloecus pictus*: (**A**) cells with prey; (**B**) egg; (**C**) young larvae; (**D**) mature larvae; and (**E**) pupae.

**Figure 5:**
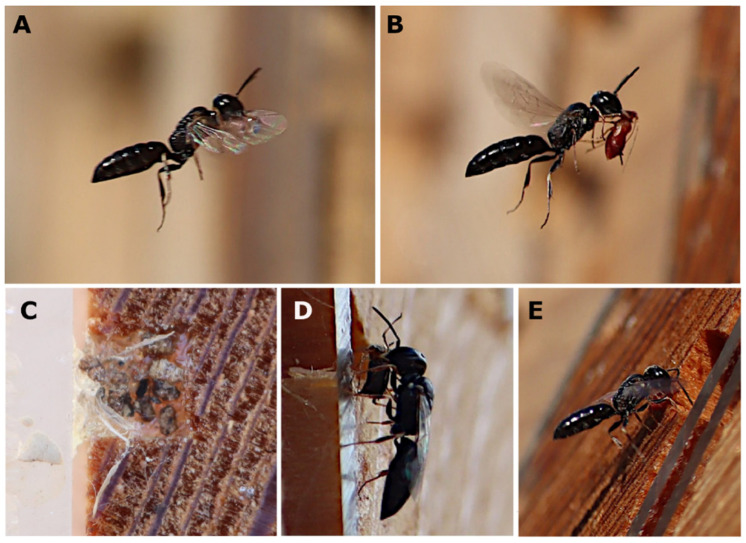
*Passaloecus pictus*: (**A**) female with resin in flight; (**B**) female with prey in flight; (**C**) nest entrances; (**D**) female with prey before entering the nest; and (**E**) female looking for wooden material to seal the tunnel entrance.

During this study, 65 trap nests were examined, including 18 tunnels occupied by *P. pictus* (Figure 2). All the nests were built between 20 June and 1 August 2024, although the peak of the nesting activity occurred in early July. The females nested only in the tunnels with a square cross-section measuring 3 × 3 × 100 mm. An analysis of all the nests showed that the sex ratio was 31:11, in favour of the females. The size of the cells in which the females developed was most often in the range of 9.5–11.0 mm × 3 mm × 3 mm, and for males it was 8–9 mm × 3 mm × 3 mm. A female stored about 33 aphids in a female cell, and 21 aphids in a male cell. An egg was usually laid on the dorsal part of an aphid. Fifty-two cells (18 tunnels) were analysed; the females developed in 31 of these cells, the males in 11 of the cells, nine cells (17.31%) were parasitised by *Melittobia acasta* (Walker, 1839) and one cell was parasitised by a chrysidid wasp. The male cells were mostly built last. The females visited a blue spruce tree (*Picea pungens* Engelm., 1879) growing within 5 m of the nest to obtain resin to separate the cells in the tunnel and seal the tunnel. The nest closures were made from a mixture of resin and particles found near the nest. No males were observed near the nests. During the day (under optimal weather conditions), the females usually provisioned two or three cells. The frequency with which the females brought aphids to the cells varied from 1 to 7 min, averaging about 2–3 min. During the inspection of the nest cells from mid to late July, almost all the cells were parasitised by *M. acasta,* and a single chrysidid wasp cocoon was found (Figure 3). The females were active from 10 a.m. to 7.30 p.m. Egg development took about three days, while larval development took about seven to nine days (Figure 4). The aphids were crushed to death in the mandibles of the wasps and carried in their mandibles to a cell prepared in advance (Figure 5). Four species of aphids were found on the plants representing the species typical of a psammophilous grassland (Table 1). The dominant prey species *Uroleucon achilleae* (Koch, 1855) [syn. *Dactynotus achilleae* (Koch, 1855)] and *Macrosiphoniella* millefolii (De Geer, 1773) were recorded in similar numbers. Other aphid species included *Dysaphis crataegi* (Kaltenbach, 1843) and *M. persequens* (Walker, 1852). The plants preferred by the aphids [15,16] were located within 2 m of the nests.

## 4. Discussion

*Passaloecus pictus* inhabits Central and Southern Europe, North Africa (Algeria), Turkey, Syria, the Caucasus, Brazil and Russia [17]. The species is most often found on the sandy, clayey and limestone slopes along rivers, at sand extraction sites, as well as in urban areas [7,9,18]. The most comprehensive studies of the biology of the genus *Passaloecus* were conducted by Janvier [8] and Tsuneki [19]. Due to the scarcity of data on the biology of the species and some discrepancies with the work of Janvier [8], we will outline a few issues below. According to the reports by Janvier [8], Ribaut [20] and Steiner [21], *P. pictus* does not nest in twigs or wood like other species of *Passaloecus*, but rather in sandstone walls, embankments and the cracks in limestone walls filled with loose clay. Our study provides evidence that the species can effectively use wooden trap nests. This fact was confirmed by Raemakers [9], in a study where the nest was located in a window frame. According to Janvier [8], the main burrow in the ground is 10–15 cm long and contains three to five brood cells. The partitions in the burrow are made of resin with the addition of plant matter or faeces [22,23], possibly as camouflage [22]. A single cell can contain up to 30 captured aphids of the genus *Macrosiphum* [8]. In our study, the number of cells per nest varied from one to a maximum of seven. The number of aphids deposited in a single cell is likely to indicate the sex of the progeny. The cells in which the females developed were usually larger and contained about 33 aphids (on average), while the cells in which the males developed contained about 21 aphids (on average). After analysing the prey and their host plants, it can be concluded that although *P. pictus* prefers amphids as food, it does not differentiate between their species and preys on the aphids existing in the vicinity. Four species of aphids were found on the plant species typical of a psammophilous grassland (Table 1). The dominant species was *Uroleucon achilleae* (syn. *Dactynotus achilleae*)—a widespread, monoecious, monophagous species found on common yarrow (*Achillea millefolium*). *Macrosiphoniella millefolii* were recorded in similar numbers—a widespread, monoecious species living on the inflorescences of yarrow (*A. millefolium*, *A. pannonica*), found less frequently on other Asteraceae such as *Anthemis arvensis* or *Chrysanthemum parthenium*. Other aphid species included *Dysaphis crataegi*—a widespread, heteroecious species, migrating from hawthorns (*Crataegus oxycantha* and *C. monogyna*) to various umbelliferous plants (Apiaceae)—and *M. persequens*—a widespread, monoecious, monophagous species found on common tansy (*Tanacetum vulgare*) [15,16]. Another related species, *P. corniger*, has been found to steal aphids from the nests of other species of the genus *Passaloecus* [24,25,26], and sometimes of the genera *Psenulus* [10,23], *P. fuscipennis* [23] and *Pemphredon*–*P. lugens* [23]. Other researchers have not observed this behaviour [27], potentially because this behaviour pays off where there is a dense population around the nest site [25]. All *Passaloecus* species provide their cells with aphids. The data on their prey are too scarce to generalise their food-source preferences, but the related genera of *Passaloecus* are not very particular and avail themselves of the nearest source of aphids [4]. The egg stage lasts three to four days [8]. A larva consumes all prey within about a week and stops feeding. It usually overwinters as a prepupa. Pupation occurs several weeks before the first flights begin [8]. Our study confirms the presence of a second generation. The nest kleptoparasites recorded to date belong to the genus *Poemenia* Holmgren, 1859 (Ichneumonidae) and the genus *Perithous* Holmgren, 1859 (Eurytomidae, Chalcidoidea), as well as to three species of Chrysididae: *Trichrysis cyanea* (Linnaeus, 1758), *Pseudomalus auratus* (Linnaeus, 1758) and *Omalus aeneus* (Fabricius, 1787) [10]. Our work is the first study to record *Melittobia acasta* in *P. pictus* nests.

## Figures and Tables

**Table 1:** Total numbers of aphids found in the nests of *Passaloecus pictus*.

Species	Number of Aphids	Main Host Plant of Aphids
*Macrosiphoniella millefolii* (De Geer, 1773) *Dysaphis crataegi* (Kaltenbach, 1843) *Macrosiphoniella persequens* (Walker, 1852) *Uroleucon achilleae* (Koch, 1855) Total	124 20 1 282 427	*Anthemis arvensis* L., *Achillea millefolium* L., *Achillea pannonica* Scheele *Anthemis arvensis* L., *Crataegus monogyna* Jacq., *Crataegus laevigata* (Poir.) DC., *Apiaceae* *Tanacetum vulgare* L. *Achillea millefolium* L.

## Data Availability

The data that support the findings of this study are available from the corresponding author upon reasonable request.
